# Cognitive Control and Emotional Intelligence: Effect of the Emotional Content of the Task. Brief Reports

**DOI:** 10.3389/fpsyg.2019.00195

**Published:** 2019-02-07

**Authors:** Purificación Checa, Pablo Fernández-Berrocal

**Affiliations:** ^1^Department of Developmental and Educational Psychology, Faculty of Education Sciences, University of Granada, Granada, Spain; ^2^Department of Basic Psychology, Faculty of Psychology, University of Málaga, Málaga, Spain

**Keywords:** emotional intelligence, cognitive processes, cool task, hot task, emotion

## Abstract

Emotional intelligence (EI) constitutes a unique form of intelligence and, from performance-based ability models, is conceptualized as the integration of several abilities: use, manage, understand, and regulate emotions. The relation between cognitive processes and EI has been less researched. Recent studies show that EI, when measured by performance-based ability models, plays a relevant role in cognitive processes when emotion is implicated in the tasks. The aim of this study was to examine the execution on hot (emotional) and cool (neutral) cognitive tasks in two groups: one high and one low on EI, in order to determine the role of EI on cognitive processes. The results showed that high and low EI groups did not differ on cool task performance, while the high EI group was better at carrying out the hot task. We discuss these results in relation to recent literature that considers the role of EI in cognitive processes.

## Introduction

Nowadays, it is assumed that cognition and emotion are two complementary aspects of the psyche, and that it is difficult to separate their influence in the performance of the activities of everyday life. From performance-based ability models, EI is defined as “the ability to perceive accurately, appraise, and express emotion; the ability to access and/or generate feelings when they facilitate thought; the ability to understand emotion and emotional knowledge; and the ability to regulate emotions to promote emotional and intellectual growth”(Mayer and Salovey, 1997). EI, as measured using ability and self-report instruments, has been linked to other factors such as work success, well-being, decision making, and stress management, among others (Joseph and Newman, 2010; Martins et al., 2010; Joseph et al., 2015; Fernández-Berrocal and Extremera, 2016; Petrides et al., 2016). In recent years, there has been increasing interest in studying how EI affects cognitive skills using laboratory tasks (Checa and Fernández-Berrocal, 2015; Gutiérrez-Cobo et al., 2017b). These tasks could be divided into ‘hot’ and ‘cool’ tasks. It is considered a ‘hot task’ when emotional stimuli or emotionally significant consequences (reward and/or loss) are used in the task. One of the most commonly used ‘hot tasks’ is the Iowa Gambling Task (IGT; Bechara et al., 1994; Kerr and Zelazo, 2004). When the stimuli used in the task are emotionally neutral, it is called a ‘cool task’. Examples of these tasks are Flanker (Eriksen and Eriksen, 1974), Go-nogo tasks, among others.

One important issue is that the approach used to obtain the EI score affects the relation found between EI and cognitive processes. EI has been found to be positively correlated with performance on hot tasks when it is evaluated by performance-based ability models (Fernández-Berrocal et al., 2014; Gutiérrez-Cobo et al., 2017b). Whether or not there is a correlation between EI and hot tasks could be discovered when self-reporting instruments or mixed models are used to evaluate EI (Pilarik and Sarmany-Schuller, 2009; Webb et al., 2014). Also, the cool tasks seem to be unrelated to EI using any EI instruments (Austin, 2005; Farrelly and Austin, 2007; Gutiérrez-Cobo et al., 2017a).

The literature suggests that EI is related to cognitive control only when the cognitive task has an emotional aspect and when EI is evaluated within performance-based ability models (Webb et al., 2014). The aim of this study was to examine whether cognitive control implemented for persons high or low on EI (groups matched on IQ) depends on the emotional content of the task. For that reason, we evaluated cognitive control on two cognitive tasks, one cool (Flanker task), more centered in the specific process of attention (suppress interfering information), and another hot (IGT), more centered in decision-making, where there are emotionally significant consequences for each EI group (low and high). We hypothesized that individuals with high EI would show a greater level of cognitive control on the hot tasks (IGT), while individuals with low EI would show no such effect. In addition, we expected that both EI groups would have similar accuracy in the cognitive control of interference on the cool task (Flanker) (Eriksen and Eriksen, 1974).

## Methods

### Participants

One hundred and seventy-eight undergraduate students from the University of Málaga completed the MSCEIT (140 women; mean age: 22 years; *SD* = 3.3 years) in order to select high and low EI participants. Based on the criterion of the mean ± 1 SD of the total scores of MSCEIT, 28 participants were selected. The high EI group included 15 participants (11 women; mean age: 22.9 years; *SD* = 4.5 years) and the low EI group included 13 participants (6 women; mean age: 22.5 years; *SD* = 2.6 years). All participants came from Spain, and their first language was Spanish. Their written and informed consent were obtained prior to participation. The study was carried out in accordance with the Declaration of Helsinki. Ethics approval was obtained from the Research Ethics Committee, University of Málaga.

### Procedure

Participants were tested at the Emotion Laboratory of the University of Málaga, Spain. The study involved two sessions of 1 h each. In the first session, participants filled in the Mayer-Salovey-Caruso Emotional Intelligence Test (MSCEIT) and the Kaufman Brief Intelligence Test (KBIT). In the second session, participants were verbally instructed on how to complete the Flanker task and IGT (described below).

### Instruments

#### Kaufman Brief Intelligence Test (KBIT)

The KBIT is an individually administered test with two subscales, Vocabulary (measure of language and experience-related knowledge) and Matrices (measure of abstract reasoning or fluid intelligence skills), as well as a composite IQ score (Kaufman and Kaufman, 2000). The Spanish version of this instrument has shown satisfactory psychometric properties (Cronbach alphas), Vocabulary α = 0.76, Matrices α = 0.82, and Composite IQ α = 0.83.

#### Mayer-Salovey-Caruso Emotional Intelligence Test (MSCEIT)

Mayer et al. (2002) is a performance-based ability measure of EI. This scale is composed of 141 items divided into four subscales according to Mayer and Salovey’s (1997): perceiving, facilitating, understanding, and managing emotions. To carry out the present study, the Spanish version of MSCEIT (Extremera et al., 2006) was used; it shows adequate psychometric properties similar to the English version (Cronbach’s α = 0.95; Sanchez-Garcia et al., 2016).

#### Hot Task (Iowa Gambling Task)

Participants were asked to choose from four decks of cards, labeled A, B, C, and D, presented on a computer screen, in order to gain play money (Bechara et al., 1994). Participants had 2000€ at the beginning of the game and were free to select one card at the time from one of four decks using a mouse. Decks A and B were Disadvantageous Decks (expected value > 0), while decks C and D were Advantageous Decks (expected value < 0). Feedback of the play money won and lost was displayed after selecting a card. The goal of the task was to gain as much play money as possible.

#### Cool Task (Flanker Task)

Each trial started with a fixation point of variable duration randomly selected between 600 and 1,200 ms (Eriksen and Eriksen, 1974). Subsequently, a target was presented until a response was made, with a maximum duration of 2,000 ms. The target display consisted of an arrow pointing either right or left that was flanked by two arrows on each side. For half the trials the flanking arrows pointed to the same (congruent) direction as the central arrow, and they pointed to the opposite (incongruent) direction for the other half of the trials (randomly assigned in each trial). Participants had to indicate the direction of the central arrow by pressing the left button for leftward pointing central arrows, and the right button for central arrows pointing right, as fast as possible. Each participant performed 192 trials divided in three blocks, with a brief break between blocks.

## Results

Behavioral results and descriptive statistics are shown in Supplementary Material: Means and standard deviations (SD) of all the dependent variables included in the study for each group. In order to determine whether cognitive control implemented for persons high or low on EI depended on the emotional content of the task, we evaluated cognitive control on two cognitive tasks, one cool (Flanker task) and the other hot (IGT), for each EI group (low and high). The two groups did not differ on scores of the two subscales, Vocabulary, *t* = -1.7, *p* = 0.10, Matrices, *t* = 0.76, *p* = 0.45 or a composite IQ score, *t* = -0.50, *p* = 0.62.

### Hot Task

The Group x Choice Type ANOVA conducted on number of choice revealed a main effect of Choice Type, *F* (1, 26) = 7.45, *p* < 0.01, η^2^ = 0.22. No significant Group x Choice Type was found, F > 1. However, a *t*-test showed that the high EI group selected more advantageous than disadvantageous choices, *t* = 5.11, *p* < 0.04, while the low EI group did not show this, *t* = 2.64, *p* < 0.13. (Supplementary Material: IGT *t*-test by block on IGT).

### Cool Task

The Group x Stimulus Type ANOVA conducted on reaction time revealed a main effect of Stimulus Type, *F*(1.26) = 33.41, *p* < 0.001, η^2^ = 0.56. No significant Group x Stimulus Type was found, *F* > 1. However, a *t*-test showed that both the high EI, *t* = 18.1, *p* < 0.001 and low EI group, *t* = 16.68, *p* < 0.002, responded faster to congruent trials than to incongruent trials.

## Discussion

The present experimental study examines whether cognitive control implemented for persons high or low on EI depended on the emotional content of the task. In controlling the effect of IQ, we found no significant differences across the two groups on all measures of IQ. We found that, consistent with our hypothesis, both groups, high and low in EI performance, had the same level on the cool task (Flanker). Both groups took more time to respond to incongruent trials than congruent ones, that is to say, both groups seemed to use similar cognitive resources to cope with interfering information. These data are consistent with the literature that shows that EI is not associated with performance on neutral cognitive tasks, when EI is measured by the MSCEIT (Farrelly and Austin, 2007). Using a similar cool task to the one used in the present study, Checa and Fernández-Berrocal (2015) did not find a relation between the ability to suppress interfering information and EI. Also, Gutiérrez-Cobo et al. (2017a) found that low and high EI groups performed equally on a go/nogo task when the task did not involve emotional information. Although these data failed to show a relation between cool cognitive tasks and EI, more investigations are needed to replicate these data.

In relation to the hot tasks, our data show that high and low EI groups differed in their performance. While low EI groups did not show a significant difference between advantage and disadvantage choices, the high EI group showed a significant difference. These data are in line with previous behavioral and ERP studies that show that EI favors cognitive performance when emotional information is needed to resolve the task (Reis et al., 2007; Fernández-Berrocal et al., 2014; Alkozei et al., 2015; Gutiérrez-Cobo et al., 2017b). A recent ERP study (Megías et al., 2017) shows that participants with high EI showed a larger N200, a brain component related to attention, than those with low EI performance on a task that involved emotional content. Using the IGT (hot task) and MSCEIT in a fashion similar to the present study (Webb et al., 2014), the study revealed that better performance on IGT was associated with higher EI, but this relation did not remain significant after controlling for IQ. In our study, high and low EI groups were equal on the IQ measure, and we found that only the high EI group showed better ability to perform on IGT. Our results are consistent with research in work settings (e.g., Joseph and Newman, 2010, p. 70) showing that, after controlling for IQ and personality, the relationship between EI and job performance was stronger for high- than for low-emotional labor jobs. Also, Alkozei et al. (2018) showed that after EI training, changes in total scores of EI measures by the MSCEIT correlated with changes in IGT performance for the EI training group. These findings suggest that EI and IQ may overlap as cognitive processes, but that EI could influence emotional tasks performance independently of IQ (Alkozei et al., 2018).

### Limitations and Future Directions

Future research should replicate these findings in a larger sample, in order to generalize it to the general population or specific population, such as gifted student. In addition, future studies can rely on these results to examine the implications for well-being, social behavior and interpersonal relations in work or educational settings.

## Author Contributions

PC was involved on acquisition of the data. PC and PF-B designed the work, analyzed and explained the data, revised the work critically for important intellectual content, and approved the version to be published.

## Conflict of Interest Statement

The authors declare that the research was conducted in the absence of any commercial or financial relationships that could be construed as a potential conflict of interest.

## References

[B1] Alkozei A. Schwab Z. J. Killgore W. D. S. (2015). The role of emotional intelligence during an emotionally difficult decision-making task. *J. Nonverbal Behav.* 40 39–54. Retrieved from 10.1007/s10919-015-0218-4

[B2] Alkozei A. Smith R. Demers L. A. Weber M. Berryhill S. M. Killgore W. D. S. (2018). Increases in emotional intelligence after an online training program are associated with better decision-making on the iowa gambling task. *Pychol. Rep.* 1 1–27. 10.1177/0033294118771705 29699472

[B3] Austin E. J. (2005). Emotional intelligence and emotional information processing. *Pers. Individ. Dif.* 39 403–414. 10.1016/j.paid.2005.01.017

[B4] Bechara A. Damasio A. R. Damasio H. Anderson S. W. (1994). Insensitivity to future consequences following damage to human prefrontal cortex. *Cognition* 50 7–15. 10.1016/0010-0277(94)90018-3 8039375

[B5] Checa P. Fernández-Berrocal P. (2015). The role of intelligence quotient and emotional intelligence in cognitive control processes. *Front. Psychol.* 6:1853. 10.3389/fpsyg.2015.01853 26648901 PMC4664650

[B6] Eriksen B. A. Eriksen C. W. (1974). Effects of noise letterls upon the identification of a target letter in a non-search task. *Percept. Psychophys.* 16 143–149. 10.3758/BF03203267

[B7] Extremera N. Fernández-Berrocal P. Salovey P. (2006). Spanish version of the mayer-salovey-caruso emotional intelligence test (MSCEIT) version 2.0: reliabilities, age, and gender differences. *Psicothema* 18 42–48. 17295956

[B8] Farrelly D. Austin E. J. (2007). Ability EI as an intelligence? Associations of the MSCEIT with performance on emotion processing and social tasks and with cognitive ability. *Cogn. Emot.* 21 1043–1063. 10.1080/02699930601069404

[B9] Fernández-Berrocal P. Extremera N. (2016). Ability emotional intelligence, depression, and well-being. *Emot. Rev.* 8 311–315. 10.1177/1754073916650494

[B10] Fernández-Berrocal P. Extremera N. Lopes P. N. Ruiz-Aranda D. (2014). When to cooperate and when to compete: emotional intelligence in interpersonal decision-making. *J. Res. Pers.* 49 21–24. 10.1016/j.jrp.2013.12.005

[B11] Gutiérrez-Cobo M. J. Cabello R. Fernández-Berrocal P. (2017a). Performance-based ability emotional intelligence benefits working memory capacity during performance on hot tasks. *Sci. Rep.* 7 1–9. 10.1038/s41598-017-12000-7 28916754 PMC5600979

[B12] Gutiérrez-Cobo M. J. Cabello R. Fernández-Berrocal P. (2017b). The three models of emotional intelligence and performance in a hot and cool go/no-go task in undergraduate students. *Front. Behav. Neurosci.* 11:33. 10.3389/fnbeh.2017.00033 28275343 PMC5319994

[B13] Joseph D. L. Jin J. Newman D. A. O’Boyle E. H. (2015). Why does self-reported emotional intelligence predict job performance? A meta-analytic investigation of mixed EI. *J. Appl. Psychol.* 100 298–342. 10.1037/a0037681 25243996

[B14] Joseph D. L. Newman D. A. (2010). Emotional intelligence: an integrative meta-analysis and cascading model. *J. Appl. Psychol.* 95 54–78. 10.1037/a0017286 20085406

[B15] Kaufman A. S. Kaufman N. L. (2000). *K-BIT, Test Breve de Inteligencia de Kaufman*. Madrid: TEA Ediciones.

[B16] Kerr A. Zelazo P. D. (2004). Development of “hot” executive function: the children’s gambling task. *Brain Cogn.* 55 148–157. 10.1016/S0278-2626(03)00275-6 15134849

[B17] Martins A. Ramalho N. Morin E. (2010). A comprehensive meta-analysis of the relationship between emotional intelligence and health. *Pers. Individ. Dif.* 49 554–564. 10.1016/j.paid.2010.05.029 21111577

[B18] Mayer J. D. Salovey P. (1997). “What is emotional intelligence?,” in *Emotional Development and Emotional Intelligence: Implications for Educators*, eds Salovey P. Sluyter D. (New York, NY: Basic Books), 3–31.

[B19] Mayer J. D. Salovey P. Caruso D. R. (2002). *Mayer-Salovey-Caruso Emotional Intelligence Test (MSCEIT) User Manual*. Toronto: MHS.

[B20] Megías A. Gutiérrez-Cobo M. J. Gómez-Leal R. Cabello R. Fernández-Berrocal P. (2017). Performance on emotional tasks engaging cognitive control depends on emotional intelligence abilities: an ERP study. *Sci. Rep.* 7 1–9. 10.1038/s41598-017-16657-y 29180769 PMC5703978

[B21] Petrides K. V. Mikolajczak M. Mavroveli S. Sanchez-Ruiz M.-J. Furnham A. Pérez-González J.-C. (2016). Developments in trait emotional intelligence research. *Emot. Rev.* 8 335–341. 10.1177/1754073916650493

[B22] Pilarik L. Sarmany-Schuller I. (2009). Emotional intelligence and decision-makinf od female students of social work in the iowa gambling task. *Stud. Psychol.* 51 319–328.

[B23] Reis D. L. Brackett M. A. Shamosh N. A. Kiehl K. A. Salovey P. Gray J. R. (2007). Emotional intelligence predicts individual differences in social exchange reasoning. *Neuroimage* 35 1385–1391. 10.1016/j.neuroimage.2006.12.045 17331743

[B24] Sanchez-Garcia M. Extremera N. Fernandez-Berrocal P. (2016). The factor structure and psychometric properties of the spanish version of the mayer-salovey-caruso emotional intelligence test. *Psychol. Assess.* 27 1–11. 10.1037/pas0000269 26751088

[B25] Webb C. A. DelDonno S. Killgore W. D. S. (2014). The role of cognitive versus emotional intelligence in iowa gambling task performance: what’s emotion got to do with it? *Intelligence* 44 112–119. 10.1016/j.intell.2014.03.008 25635149 PMC4307614

