# Supplementary material for: Cognitive Control and Emotional Intelligence: Effect of the Emotional Content of the Task. Brief Reports
Source: Front Psychol. 2019 Feb 7;10:195. doi: 10.3389/fpsyg.2019.00195 (PMC6374306; doi:10.3389/fpsyg.2019.00195)
Supplement: Supplementary file 1 [file Data_Sheet_1.PDF]

Supplementary material.

Means and standard deviations (SD) of all the dependent variables included in the study for each group

		Mean	(SD)	Mean	(SD)	
**Task**	**Group**	**Dependent Variables**				
**Hot (IGT)**		**ADV**		**DIS**		**T-test**
	High EI	57	(11.9)	43	(11.9)	**5.12***
	Low EI	54	(9.5)	46	(9.5)	2.64
**Cool (Flanker)**		**CON**		**INC**		
	High EI	406.1	(45.8)	430.2	(46)	**18.05****
	Low EI	398.2	(36.9)	417.8	(33.2)	**16.68****

ADV = advantage choices; DIS= disadvantage choices; CON= RT congruent trial; INC=RT incongruent trials
Significance level: * $p < .05$; ** $p < .01$

T-test by block on IGT

		Mean	(SD)	Mean	(SD)	
**Group**		**Dependent Variables**				
High EI		**ADV**		**DIS**		**T-test**
	**Block 1**	10	5.3	10	**5.3**	-.23
	**Block 2**	12	3.6	8	**3.6**	**2.01#**
	**Block 3**	12	2.6	8	**2.6**	**1.94#**
	**Block 4**	11	3.9	9	**3.9**	**2.10#**
	**Block 5**	12	3.2	8	**3.2**	**2.06#**
Low EI						
	**Block 1**	11	4.6	9	**4.6**	.52
	**Block 2**	10	2.7	10	**2.7**	.86
	**Block 3**	11	3.6	9	**3.6**	1.56
	**Block 4**	10	2.3	10	**2.3**	.28
	**Block 5**	11	3.3	9	**3.3**	.97

ADV = advantage choices; DIS= disadvantage choices;
Significance level: # $p < .10$
